# Bismuth-Doped Nanohydroxyapatite Coatings on Titanium Implants for Improved Radiopacity and Antimicrobial Activity

**DOI:** 10.3390/nano9121696

**Published:** 2019-11-27

**Authors:** Gabriela Ciobanu, Maria Harja

**Affiliations:** Faculty of Chemical Engineering and Environmental Protection “Cristofor Simionescu”, “Gheorghe Asachi” Technical University of Iasi, Prof. dr. docent Dimitrie Mangeron Rd., no. 73, 700050 Iasi, Romania

**Keywords:** bismuth-doped nanohydroxyapatite, coating, titanium implant, radiopacity, antimicrobial activity

## Abstract

This study aims to present the possibility to obtain bismuth-doped nanohydroxyapatite coatings on the surface of the titanium implants by using a solution-derived process according to an established biomimetic methodology. The bioactivity of the titanium surface was increased by an alkali-thermal treatment. Then, under biomimetic conditions, the titanium surface was coated with a Bi-doped nanohydroxyapatite layer by using a modified supersaturated calcification solution (SCS) containing a bismuth salt. The apatite deposits were analyzed by scanning electron microscopy coupled with X-ray analysis (SEM-EDX), X-ray photoelectron spectroscopy (XPS), X-ray diffraction (XRD), and digital X-rays radiography method. The results indicate that the Bi-doped nanohydroxyapatite coatings on titanium surface were produced. These coatings exhibit a good radiopacity, thus enhancing their applications in dental and orthopedic fields. Additionally, the Bi-doped nanohydroxyapatite coatings show significant antimicrobial activity against *Escherichia coli* and *Staphylococcus aureus* bacteria.

## 1. Introduction

Among the materials available for implant applications in dental and orthopedic restoration, titanium and its alloys are largely used due to their special characteristics such as high corrosion resistance, superior mechanical properties, and good biocompatibility [1].

The use of Ti implants in reconstructive surgery is in many cases affected by some Ti surface properties, such as wear, hardness, and mainly slow osseointegration. Therefore, diverse methods have been developed to improve surface properties of Ti implants, such as morphological modifications (regarding roughness, morphology, etc.) by mechanical, chemical, and physical methods, or deposition of organic or inorganic coatings on the Ti surface [2,3]. Many studies indicate that Ti implants can be coated with calcium phosphate (e.g., hydroxyapatite) layers by various deposition methods such as electrodeposition, plasma spray, high-frequency magnetron sputtering, biomimetic precipitation, etc. [4,5,6,7,8]. In this way, the bioactivity, biocompatibility, and corrosion resistance of the Ti-based implants are improved.

The hydroxyapatite Ca_10_(PO_4_)_6_(OH)_2_ is a calcium phosphate ceramic found in nature, but can be obtained by various methods. In the human body, it is the main inorganic component of bone and dental dentin and enamel [9]. Synthetic hydroxyapatite can be obtained by various methods, the most used being wet methods (chemical precipitation, sol-gel, hydrothermal, biomimetic, or electrodeposition) [10]. Hydroxyapatite has many medical applications, especially as a basic dental or bone material in reconstruction and repair surgery. The use of hydroxyapatite as a bone substitute material is unfortunately limited due to its poor mechanical properties, especially its brittleness and inflexibility. Therefore, many studies have focused on obtaining polymeric or metal composite implants containing hydroxyapatite in order to improve the osseointegration process [11,12]. Additionally, the crystal structure of the hydroxyapatite allows the substitution of Ca^2+^ ions with different foreign ions (Na^+^, Zn^2+^, Mg^2+^, Ce^3+^, Y^3+^, Ti^4+^, etc.) in small quantities and this substitution can increase the osteoblast adhesion and enhance the properties of the hydroxyapatite as biomaterial with medical applications [13,14,15,16,17].

The bismuth (Bi) compounds, especially Bi(III), are widely applied in catalysis, pharmaceutical, and medical fields. The main medical applications of bismuth compounds are noticed in radiographic, anticancer, and antimicrobial studies [18,19]. Some bismuth salts are used for medical purposes to treat gastrointestinal, infectious, or dermatological diseases. Additionally, bismuth alloys may be used in the realization of bone and dental devices. Materials containing bismuth show high radiopacity and can thus be used as contrast materials in bone and dental restorative cements to obtain better imaging information in X-ray analysis (e.g., computed tomography).

Studies on hydroxyapatite doped with bismuth are very few [20,21]. In our previous investigations we have demonstrated the possibility of synthesizing Bi-doped hydroxyapatite nanopowders [22,23]. Instead, the Bi-doped nanohydroxyapatite deposited as a thin film on the surface of the titanium implants has not been studied before by anyone. 

Therefore, this research presents the possibility to obtain, by a biomimetic technique, Bi-doped nanohydroxyapatite coatings on the surface of the titanium implants. A supersaturated calcification solution (SCS) modified by adding an appropriate quantity of bismuth salt was used to achieve biomimetic Bi-doped nanohydroxyapatite coatings. By dipping a titanium sample in the SCS solution, the hydroxyapatite nucleation on the titanium surface takes place in a short time, which then grows over time uniformly covering the metallic surface. By adding an additional source of bismuth to the SCS solution, the incorporation of bismuth ions into the hydroxyapatite lattice is facilitated. These Bi-doped nanohydroxyapatite coatings were characterized and tested for their radiopacity and bactericidal behavior.

## 2. Materials and Methods 

### 2.1. Materials

Calcium chloride dihydrate (CaCl_2_∙2H_2_O), monosodium phosphate monohydrate (NaH_2_PO_4_∙H_2_O), sodium bicarbonate (NaHCO_3_), bismuth (III) nitrate pentahydrate (Bi(NO_3_)_3_∙5H_2_O), sodium hydroxide (NaOH), ethanol and acetone were acquired from Sigma-Aldrich (Germany) and used without further purification.

### 2.2. Coating Solutions

In this study, certain amounts of CaCl_2_∙2H_2_O, NaH_2_PO_4_∙H_2_O, and NaHCO_3_ reagents were dissolved in 1 L of deionized water, under vigorous stirring, in order to obtain the supersaturated calcification solution (SCS), as presented elsewhere [24]. In this solution, the ion concentrations were of 6.5 mmol/L Na^+^, 10 mmol/L Ca^2+^, 20 mmol/L Cl^−^, 5 mmol/L H_2_PO_4_^−^, and 1.5 mmol/L HCO_3_^−^, and the Ca/P atomic ratio was 1.67 (Table 1), as in biological hydroxyapatite [9].

By adding a certain amount of Bi(NO_3_)_3_∙5H_2_O salt to the original SCS solution, the modified SCS solution (denoted Bi-SCS) was obtained. In this solution, the (Bi + Ca)/P and x_Bi_ = Bi/(Bi + Ca) atomic ratios were of 1.67 and 0.01, respectively (Table 1). The concentration of Bi in Bi-SCS solution is low (of about 1%), according to the therapeutic range mentioned in literature [25].

### 2.3. Alkali-Thermal Treatment

The plates of commercially pure Ti (c.p. Ti) of 10 × 10 × 3 mm in size were polished using silicon carbide (SiC) paper and then cleaned in an ultrasonic bath with distilled water. Prior to alkali-thermal treatment all the samples were cleaned for 15 min in acetone, 10 min in ethanol, and 5 min in deionized water. Then, all samples were subjected to an alkaline treatment in 0.6 M NaOH solution at 160 °C in a pressure chamber for 24 h, at heating rates of 5 °C/min. Finally, the samples were rinsed for 5 min in deionized water and then activated by a thermal oxidation treatment at 600 °C for 3 h in a furnace with a heating rate of 5 °C/min.

### 2.4. Biomimetic Deposition

After alkali-thermal treatment, the Ti samples were subjected to a biomimetic treatment in SCS (or Bi-SCS) solution at 37 °C, as presented elsewhere [24]. Periodically, these biomimetic solutions (SCS or Bi-SCS) were refreshed in order to keep the ion concentrations constant. After a certain period of time, the Ti samples were removed from the biomimetic solution (SCS or Bi-SCS), rinsed with deionized water, and then dried in an oven for 1 h at 37 ° C.

The Ti samples covered with undoped hydroxyapatite and Bi-doped hydroxyapatite layers were denoted *HA-Ti* and *Bi-HA-Ti*, respectively.

### 2.5. Sample Characterization

Scanning electron microscopy (SEM) measurements were performed using a QUANTA 200 3D microscope (FEI, Eindhoven, The Netherlands), equipped with an energy dispersive X-ray spectrometer (EDX).

X-ray photoelectron spectroscopy (XPS) measurements were performed using a PHI-5000 VersaProbe photoelectron spectrometer (Φ ULVAC-PHI, INC., Chigasaki, Japan), equipped with a hemispherical energy analyzer (0.85 eV binding energy resolution). A monochromatic Al Kα X-ray radiation (hν = 1486.7 eV) was used as excitation source, operating at 15 kV and 20 mA.

X-ray diffraction (XRD) measurements were performed using a X’PERT PRO MRD diffractometer (PANalytical, Almelo, The Netherlands), with CuKα (λ = 0.15418 nm) radiation, operating at 40 kV and 50 mA over a 2θ range from 2 to 70°.

The radiographs of the samples were obtained in a dental X-ray system (X-Mind™ AC, SATELEC, Mérignac, France). The samples were placed on an occlusal radiographic film and exposed along with a graduated aluminum (99.5% pure) step wedge with thicknesses varying from 1 to 10 mm in 1 mm increments. The radiographs were digitized using a desktop scanner (VistaNet/VistaScan PERIO PLUS, Bietigheim-Bissingen, Germany). The digitized images were then imported into the Gendex Dental Systems VixWin 2000 software ((1.11 /17 Apr 2005 version, Gendex Dental Systems Manufacturer, Des Plaines, IL, USA) where a tool was applied to identify equal-density areas in the radiographic images. The areas of the aluminum step wedge and the samples were selected to determine the radiopacity values of the samples which were expressed in terms of equivalent millimeters of aluminum (mm Al). Three measurements were made for each cited area and the means of these readings calculated.

### 2.6. Antibacterial Tests

The antibacterial activity of the samples was investigated against Gram-positive *Staphylococcus aureus* and Gram-negative *Escherichia coli* bacterial strains, by using standardized Kirby–Bauer disc diffusion method [26]. The samples were planted in a Mueller–Hinton agar inoculated with *Escherichia coli* or *Staphylococcus aureus* bacteria, followed by incubation at 37 °C for 24 h. To evaluate the antibacterial activity of the samples, the total diameter (in mm) of the inhibition zone was measured for each sample. The antibacterial assessment was performed in duplicate and the average results were reported. The values are expressed as means ± standard deviations. Statistical analysis was performed using Student’s *t*-test, with the significant level with a *p* value of less than 0.05.

## 3. Results and Discussion

### 3.1. Mechanism of Coating Formation

In this study, the Ti samples were subjected to two consecutive treatments: (i) an alkali-thermal treatment, and (ii) a biomimetic treatment.

During alkali treatment, the hydroxyl groups (into alkaline solution) partially dissolve the passive TiO_2_ layer on the titanium surface, leading to the formation of the negatively charged hydrates (HTiO_3_^−^∙nH_2_O) [27]. These negatively charged species interact with the Na^+^ ions in the aqueous solution leading to the formation of a sodium titanate hydrogel layer which is transformed into crystalline sodium titanate (Na_2_Ti_5_O_11_), after the heat treatment.

After alkali-thermal treatment, the undoped or Bi-doped hydroxyapatite coatings on the surface of the titanium implant were obtained by biomimetic method, using a supersaturated calcification solution (SCS or Bi-SCS) at 37 °C, mimicking the physiological conditions [28].

In the biomimetic SCS solution, the Na^+^ ions from the sodium titanate layer on the Ti surface are replaced by H_3_O^+^ ions via ion-exchange mechanism, leading to the formation of negatively charged Ti-OH^−^ groups on the Ti surface [28]. The Ti-OH^−^ groups electrostatically attract the positively charged Ca^2+^ ions in the SCS solution forming calcium titanate, which in turn takes the PO_4_^3−^ ions in the solution to form apatite nuclei on the Ti surface. Hydroxyapatite nuclei grow over time consuming calcium and phosphate ions from the SCS solution, thus forming a hydroxyapatite layer on the entire titanium surface.

In the modified biomimetic Bi-SCS solution containing Bi^3+^ ions, the Bi-doped hydroxyapatite layer is formed on the Ti surface by a mechanism similar to that into the SCS solution, with the difference that the titanate layer also retains the Bi^3+^ ions in the solution together the PO_4_^3−^ and Ca^2+^ ions to form the Bi-doped hydroxyapatite crystals. During the synthesizing process, the incorporation of the Bi^3+^ ions into the hydroxyapatite structure via Ca^2+^↔Bi^3+^ substitution mechanism is favorable because the ionic radii of the two ions are very close in size, according to Shannon [29].

### 3.2. Coating Morphology and Structure

**Figure 1:**
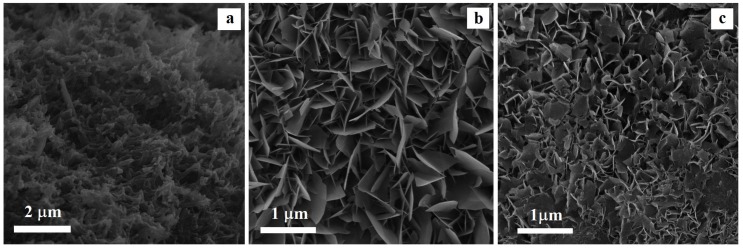
Scanning electron microscopy (SEM) photographs of the titanium surface after alkali-thermal treatment (**a**) and after soaking in SCS (**b**) or in Bi-SCS (**c**) solutions (for 72 h at 37 °C).

The SEM photographs presented in Figure 1 show the microstructure evolution of the titanium surface after alkali-thermal treatment and biomimetic treatment in a supersaturated calcification solution (SCS or Bi-SCS).

As can be seen in Figure 1a, after alkali-heat treatment on the titanium surface a needle-shaped structure was formed, in good agreement with several researchers which have reported such sodium titanate morphology [28,30]. The corrosive attack of NaOH on the Ti surface leads to the growth of a porous layer with sub-micrometric porosity, free of cracks, and uniformly covering the entire sample surface. The XRD investigation (figure not shown) pointed out that sodium titanate (Na_2_Ti_5_O_11_) is formed on the Ti surface.

As shown in Figure 1b, after 72 h of immersion into SCS solution, the Ti surface was completely covered by a continuous layer of hydroxyapatite, as confirmed by XRD and EDX analyses. The formation of hydroxyapatite particles with plate-like shape agrees with the results of several works by using simulated body fluids [31,32].

In the Bi-SCS solution, a Bi-doped hydroxyapatite layer was deposited on metallic substrates (Figure 1c). According to the XRD data the presence of Bi^3+^ ions reduced the dimensions of the particles of plate-like shape from micron to the nanometer level. This result indicates the presence of Bi^3+^ ions to inhibit the hydroxyapatite crystals from growing, leading to the formation of nanohydroxyapatite coatings.

**Figure 2:**
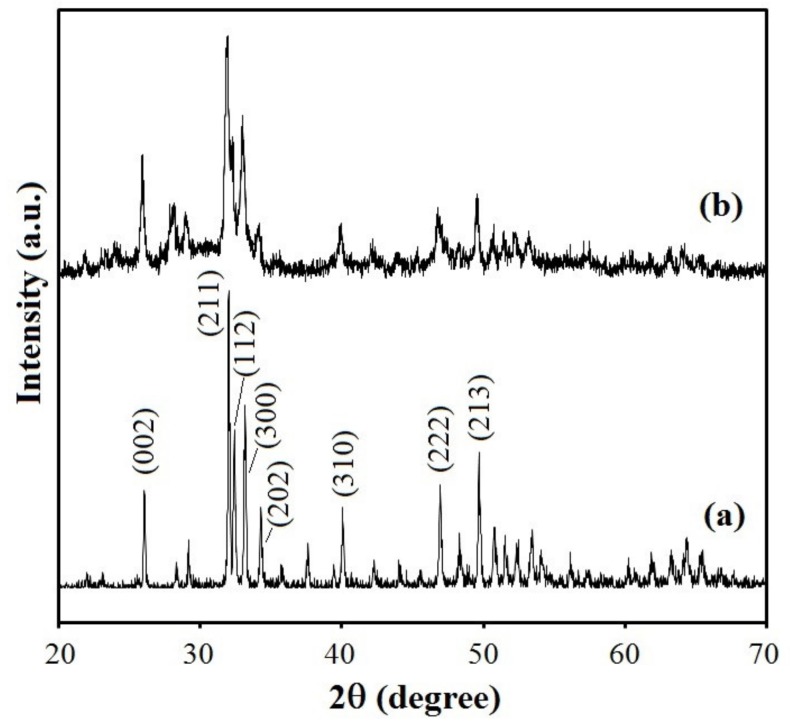
XRD spectra of the hydroxyapatite coatings deposited on the titanium surface after soaking in SCS (**a**) or in Bi-SCS (**b**) solutions (for 72 h at 37 °C).

The XRD patterns of the undoped and Bi-doped hydroxyapatite layers on the Ti surface are presented in Figure 2, indicating an overall crystalline nature of the products. According to the phase analysis, samples are in good agreement with the hexagonal (space group P63/m) hydroxyapatite phase (JCPDS Data Card 09–0432). In comparison with undoped hydroxyapatite layer, the Bi-doped hydroxyapatite layer showed similar peaks without significant shifting of the peak positions. The lattice parameters of the undoped HA and Bi-doped hydroxyapatite layers on Ti surface obtained from the DRX spectra have very close values: a = 9.5297 Å and c = 6.8769 Å for undoped HA, and a = 9.5271 Å and c = 6.8333 Å for Bi-doped hydroxyapatite. Therefore, the Bi^3+^ ions were incorporated into the crystalline network of the hydroxyapatite without greatly altering its structure, as confirmed by XPS analyses. However, in comparison with undoped hydroxyapatite layer, the XRD pattern of the Bi-doped hydroxyapatite layer shows broader and less intense peaks indicating a decrease in crystallinity. This decrease could be attributed to a different charge compensation mechanism for Ca^2+^↔Bi^3+^ isomorphous substitution. The decreased crystallinity might further lead to increased solubility, thus contributing to the local release of bismuth ions which may in turn improve the biodegradability and antibacterial properties. The broad diffraction peaks indicate also that the particles consist of smaller crystallites in nanometric size, as shown in the SEM image (Figure 1c).

### 3.3. Coating Chemical Composition

**Figure 3:**
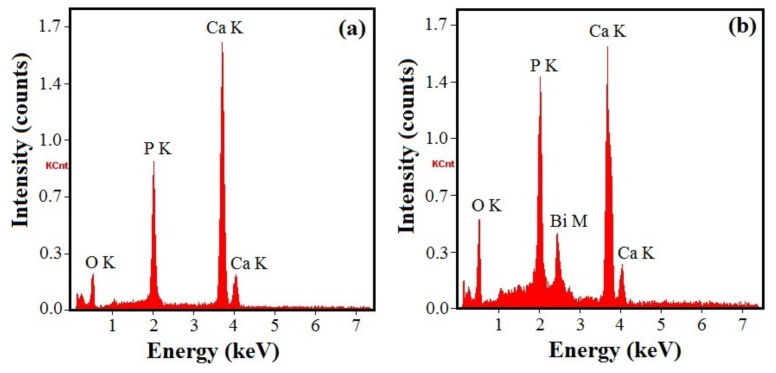
Energy dispersive X-ray spectrometer (EDX) spectra of the hydroxyapatite coatings deposited on the titanium surface after soaking in SCS (**a**) or in Bi-SCS (**b**) solutions (for 72 h at 37 °C).

The EDX spectra of the undoped and Bi-doped hydroxyapatite layers on Ti surface are presented in Figure 3, indicating the existence of the characteristic peak of bismuth for the last. All the samples contain calcium or/and bismuth, phosphorous, oxygen, and hydrogen in certain contents. The mass fractions of the different elements in the undoped-hydroxyapatite (HA-Ti sample) and Bi-doped hydroxyapatite (Bi-HA-Ti sample) layers were obtained and the atomic ratios calculated as shown in Table 1. The Ca/P or (Bi + Ca)/P atomic ratios of the formed apatite coatings were found to be around 1.67 similar to the stoichiometric value of hydroxyapatite as mentioned in literature [9,33]. The EDX studies confirm the purity of the prepared hydroxyapatite and bismuth-doped hydroxyapatite coatings containing Ca_10-x_Bi_x_(PO_4_)_6_(OH)_2_ with x_Bi_ = 0 (HA-Ti sample) and x_Bi_ = 0.0098 (Bi-HA-Ti sample). Therefore, the results suggest that the Bi^3+^ ions were successfully incorporated into the hydroxyapatite crystalline network deposited on the Ti surface, the hydroxyapatite being a suitable matrix for incorporating Bi^3+^ ions.

**Figure 4:**
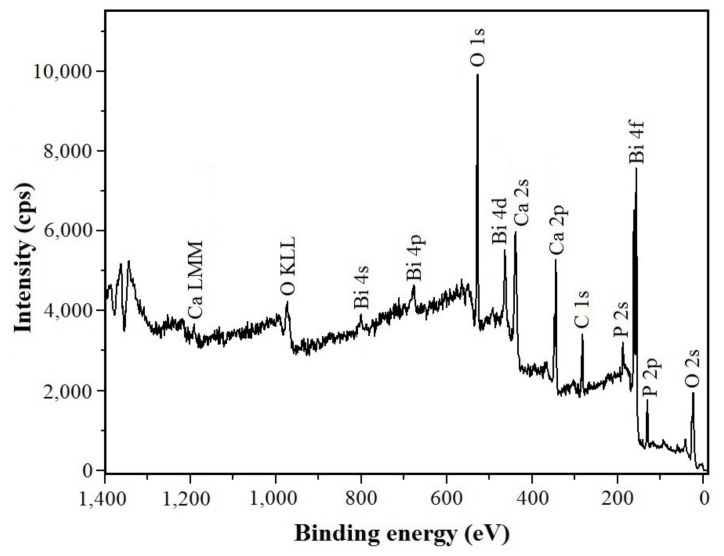
X-ray photoelectron spectroscopy (XPS) spectrum of the Bi-doped hydroxyapatite layer deposited on the titanium surface after soaking in Bi-SCS solution (for 72 h at 37 °C).

In Figure 4 the XPS spectrum of the Bi-doped hydroxyapatite layer obtained after 72 h soaking into Bi-SCS solution containing Bi^3+^ ions is depicted. The binding energies of Ca (2p, 345.1 eV), O (1 s, 528.9 eV), P (2p, 131.1 eV), and also of Bi (4f region, 150–170 eV; peaks at 156.9 and 162.2 eV) were detected. Consequently, the XPS data demonstrate that the hydroxyapatite lattice contains Bi^3+^ ions. No signal for Ti metal was noticed which indicates that the apatite layer fully covers the surface. The C 1s (282.6 eV) signal is due to the carbon used as an internal reference. No other impurities were evident in the samples in good agreement with the XRD data.

### 3.4. Radiopacity

Dental fillings, cements, ceramics, metals, and bone graft materials have to show a relative degree of radiopacity for being radiologically distinguished, depending on the radiopacity of their surrounding and/or neighboring hard and soft tissue structures. The radiopacity of these materials must be higher than that of dentin (with radiopacity of about 2.5 mm Al), which is the acceptable inferior limiting value of the radiopacity [34]. If the material presents radiopacity similar to or lower than that of dentin, recognition of the faulty proximal contour is impaired, as well as the diagnosis of some defects that lead to clinical failure. Many authors suggest that a radiopacity value higher than that of enamel (with radiopacity of about 4.1 mm Al) is desirable in order to detect restorative osseous materials [35].

**Figure 5:**
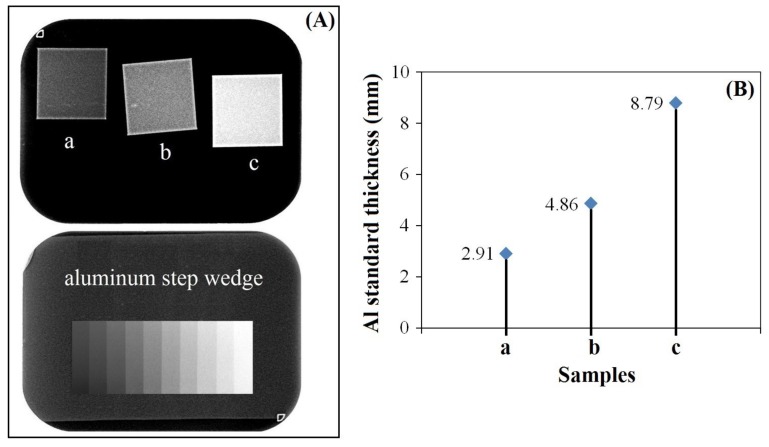
X-ray photographs (**A**) and corresponding grayscale value graph (**B**) of the titanium surface after alkali-heat treatments (a) and after soaking in SCS (b) or in Bi-SCS (c) solutions. The aluminum standards are shown in (**A**) (bottom).

In the present study, the Bi-doped nanohydroxyapatite layer was formed on the titanium surface and the contrast enhancement provided by the substituted nanohydroxyapatite was evaluated through X-ray imaging. For comparison, the hydroxyapatite layers without bismuth ions and with only 1% Bi^3+^ ion substitution were produced and evaluated for their contrast properties. The X-ray radiographical images of the undoped and Bi-doped hydroxyapatite layers coated on titanium surface are shown in Figure 5. Figure 5B shows the grayscale value graph in which the contrast was quantified with respect to the thickness of aluminium standards (1–10 mm). As we can clearly observe, nanohydroxyapatite coating doped with only 1% Bi^3+^ ions exhibited more radiopacity than the undoped hydroxyapatite and the sodium titanate. Moreover, we can appreciate the homogeneity of the samples in the photographs. When evaluating the radiopacity of the samples compared to the radiopacity of enamel [35], only Bi-substituted nanohydroxyapatite layer coated on titanium surface had superior radiopacity. These results indicate that Bi-substituted nanohydroxyapatite layers on titanium implants can be candidates as a contrast medium in terms of their radiopacity for X-ray imaging.

### 3.5. Antibacterial Activity

An ideal biomaterial should show some antimicrobial capacity to protect the tissues from secondary infection caused by residual bacteria or microleakage.

Synthetic hydroxyapatite alone has no or very little antimicrobial activity [36]. The introduction of a small number of foreign ions (Ag^+^, Cu^2+^, Zn^2+^, Sr^2+^, Ce^3+^, Ga^3+^, Ti^4+^, and others) with antibacterial properties in the apatite structure can improve its antimicrobial activity [37].

The antibacterial properties of the hydroxyapatite and Bi-substituted nanohydroxyapatite layers on titanium implants were performed through the inhibition zone method against Gram-positive *Staphylococcus aureus* and Gram-negative *Escherichia coli* bacteria. These bacteria were chosen because most bone infections are of bacterial origin, and approximately half of them are monomicrobial. Staphylococci, in particular *Staphylococcus aureus*, are the predominant cause of bone infections worldwide. Other aerobic bacteria that may be involved include *Escherichia coli* [38,39]. Bacteria or their products can directly increase osteoclast formation and activity, and the inflammatory environment at sites of infection can further promote bone resorption [40].

**Figure 6:**
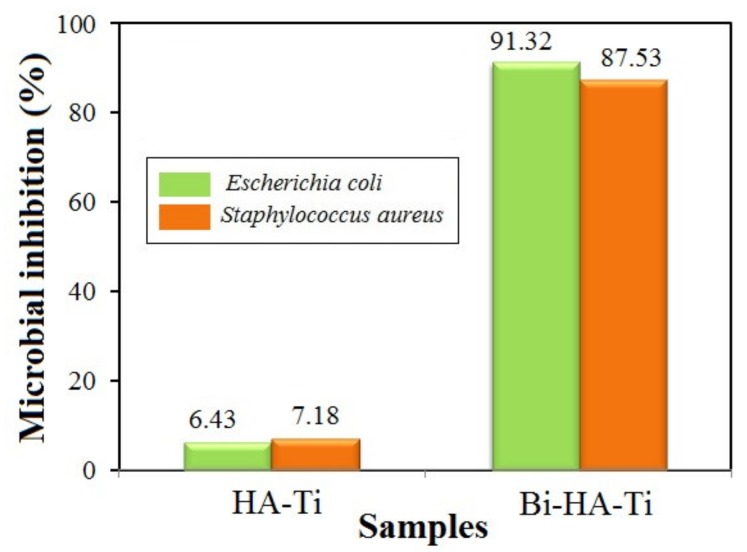
Microbial inhibition of the undoped and Bi-doped hydroxyapatite layers deposited on titanium surface against *Escherichia coli* and *Staphylococcus aureus* bacteria.

As revealed in Figure 6, the level of microbial growth of both bacteria varies differently in the Bi-doped nanohydroxyapatite layer compared to the undoped hydroxyapatite layer. The antibacterial property against *Escherichia coli* and *Staphylococcus aureus* is improved after Ca^2+^↔Bi^3+^ substitution into the hydroxyapatite lattice. This can be explained by the better solubility of the Bi-doped nanohydroxyapatite than that of pure hydroxyapatite, which releases bismuth ions to inhibit the proliferation of tested bacteria.

Based on the obtained results, the Bi-doped nanohydroxyapatite coatings on the titanium surface could be considered as promising antimicrobial agents due to their bioactive properties.

## 4. Conclusions

A biomimetic method was applied for the deposition of the Bi-doped nanohydroxyapatite coatings on the pure Ti implant surface. Alkali-thermal treatment of the Ti surface created better treatment conditions for obtaining a bioactive material. The nanohydroxyapatite doped with a small concentration of bismuth (1%) was coated on a Ti implant surface by using a supersaturated calcification solution (Bi-SCS) modified by adding a bismuth salt. Bismuth was found to incorporate into the apatite layer via the Ca^2+^↔Bi^3+^ substitution mechanism into the apatite lattice. The presence of Bi^3+^ ions in Bi-SCS solution inhibits the hydroxyapatite crystals from growing, thus leading to the formation of nanohydroxyapatite coatings. The Bi-doped hydroxyapatite coating with 1% bismuth showed higher radiopacity than the undoped hydroxyapatite. The results also demonstrate that Bi-doped hydroxyapatite coating possesses superior antibacterial activity against *Escherichia coli* and *Staphylococcus aureus* bacteria compared to the undoped hydroxyapatite coating. The in vitro antibacterial tests demonstrate that titanium implants with Bi-doped nanohydroxyapatite on the surface might be useful for better infection control. The results support the use of coating titanium dental implant surfaces with Bi-doped nanohydroxyapatite to provide a radiopacity and antimicrobial function.

## Figures and Tables

**Table 1:** Atomic ratios in the supersaturated calcification solution (SCS) and in the final coatings.

Sample	SCS Solution			Final Coating		
	$x_{Bi}=\frac{Bi}{Bi+Ca}$	$\frac{Bi+Ca}{P}$	Bi (%)	$x_{Bi}=\frac{Bi}{Bi+Ca}$	$\frac{Bi+Ca}{P}$	Bi (%)
HA-Ti	0	1.677	0	0	1.673	0
Bi-HA-Ti	0.01	1.677	1	0.0098	1.671	0.98
